# Unseen contaminants in Portuguese reservoirs: linking microplastics to ecological potential and human pressures

**DOI:** 10.3389/ftox.2025.1705228

**Published:** 2025-11-27

**Authors:** C. Guimarães, I. Pinto, J. A. Padilha, S. C. Antunes

**Affiliations:** 1 DBio FCUP - Departamento de Biologia, Faculdade de Ciências da Universidade do Porto, Porto, Portugal; 2 CIIMAR/CIMAR LA, Centro Interdisciplinar de Investigação Marinha e Ambiental, Universidade do Porto, Matosinhos, Portugal; 3 ICBAS - Instituto de Ciências Biomédicas de Abel Salazar, Universidade do Porto, Porto, Portugal; 4 UMIB - Unidade Multidisciplinar de Investigação Biomédica - Instituto Ciências Abel Salazar da Universidade do Porto, Porto, Portugal; 5 CBMA – Centro de Biologia Molecular e Ambiental/ARNET - Rede de Pesquisa Aquática e IB-S, Instituto de Ciência e Inovação para Biosustentabilidade, Departamento de Biologia, Universidade do Minho – Campus de Gualtar, Braga, Portugal

**Keywords:** emerging pollutants, ATR-FTIR analysis, water quality, Water Framework Directive, heavily modified water bodies, lentic ecosystems

## Abstract

The accumulation of microplastics (MPs) in aquatic environments is a contemporary concern of great relevance, however, freshwater ecosystems, particularly reservoirs, have received less attention. This study evaluates the MPs in Rabagão and Aguieira Portuguese reservoirs, and their role in ecological quality assessments. Along 2023, sub-surface water samples were collected to assess Ecological Potential, under Water Framework Directive (WFD) metrics, and to characterize MPs by type, colour, size, and chemical composition. Reservoirs were also characterized by land use, soil occupation, and anthropogenic pressures. Results confirm MPs contamination in both reservoirs, predominantly fibres, with Rabagão exhibiting higher total abundance (Rabagão 5,862 *vs* Aguieira 1,658 MPs). Microplastic concentrations varied across sampling sites and periods in both reservoirs, with the Rabagão reservoir exhibiting greater spatial variation among sites within sampling periods and more pronounced seasonal fluctuations. In both study areas, the highest abundances were consistently recorded near the dams. In both reservoirs, the predominant colours were blue, black, and grey, and the most observed size ranged from 0.1 to 0.5 mm. ATR-FTIR analysis identified polyethylene, polyethylene terephthalate, polyester, nylon, polyvinyl chloride, and polyvinyl acrylate. Anthropogenic pressures including aquaculture, wastewater discharges, and recreational activities were identified as potential pollution sources. Despite fewer pressures and better ecological status (according to the parameters evaluated following the WFD approach), Rabagão had higher microplastic contamination. On the contrary, Aguieira, which exhibited poorer ecological quality, had lower microplastic concentrations. This finding emphasizes that conventional water quality indicators may not adequately reflect the presence and influence of MPs, reinforcing the need to incorporate them into ecological assessment frameworks, especially in reservoirs used for human purposes.

## 1 Introduction

Nowadays, plastic pollution is recognized as one of the most concerning environmental issues, as it has large-scale impacts and can spread from terrestrial to aquatic ecosystems through runoff, leachate, effluent discharges, or even wind (Borrelle et al., 2020; Häder et al., 2020). Plastics are human-made materials widely used due to their versatility, durability, and low cost (Bertoldi et al., 2021), and an exponential rise in production has been recorded, reaching 413.8 million tons in 2023 worldwide (Plastics Europe, 2024). However, with only 9% of plastic recycled globally (OECD, 2022), plastic waste is accumulating in the environment, impacting a wide range of ecosystems, with plastics now ubiquitous in aquatic, terrestrial, and even remote regions like the deep ocean, the Arctic, and the atmosphere (Akanyange et al., 2022). As plastic waste undergoes slow degradation and fragmentation processes, it releases toxic substances, such as heavy metals and persistent organic pollutants (Ali et al., 2021; Liu L. et al., 2022). This degradation generates both secondary microplastics (MPs) and nanoplastics, which result from the breakdown of larger plastic items, and primary MPs, which are small plastic particles intentionally manufactured and released directly into the environment (Zhang et al., 2015; Jiang et al., 2020; Bajt, 2021).

A significant portion of MPs ends up in aquatic ecosystems, primarily originating from terrestrial sources, particularly the cosmetic and hygiene products industry, which extensively uses MPs in items like facial exfoliants and toothpaste. The textile industry also uses synthetic fibres, such as polyester, nylon, and acrylic, which are continuously released during laundry (Osman et al., 2023; Periyasamy, 2023). Conventional wastewater treatment plants (WWTPs) are unable to fully retain these pollutants, resulting in their discharge into rivers (Golwala et al., 2021). Additional sources of MPs include ship cleaning and maintenance and tire wear, which introduce plastic particles into aquatic environments through runoff or wind (Boucher and Friot, 2017). Agriculture contributes to the issue as well, particularly through practices like plastic mulching, as mentioned by Kallenbach et al. (2022). Another portion of the MPs present in aquatic ecosystems results from activities that occur within those ecosystems, including commercial and recreational fishing, aquaculture, and naval tourism, which release various forms of MPs, such as paint fragments from ships and boats and fishing net fibres (Tamburri et al., 2022; Kuroda et al., 2024).

Once in the aquatic environment, MPs can contribute to biodiversity loss and ecosystem degradation, as demonstrated by several studies (Ma et al., 2020; Xu et al., 2020; Tu et al., 2023). Due to their small size, MPs can be accidently ingested by invertebrates and fish, leading to harmful effects, including inflammation and chemical toxicity (Benson et al., 2022). Furthermore, these particles can accumulate through the food web, eventually reaching humans and posing potential health risks (Li et al., 2020). Additionally, certain physical and chemical properties of MPs (e.g., high surface-area-to-volume ratio, nonpolar surfaces) promote the adsorption of contaminants onto their surface, allowing MPs to act as vectors for toxic substances that can be ingested by organisms or introduced into ecosystems (Benson et al., 2022; Liu L. et al., 2022). Due to their harmful effects on flora and fauna, high abundance from continuous release, and resistance to degradation, MPs are now considered emerging pollutants in the aquatic environment (Fu et al., 2021; Hube and Wu, 2021). Consequently, studying the occurrence, distribution, sources, and impacts of MPs in aquatic ecosystems has become a key focus of current scientific research (Auta et al., 2017). While much of the focus has been on marine environments (Ferreira et al., 2023; Alomar et al., 2024), recent studies are increasingly addressing freshwater ecosystems, such as rivers, which play a significant role in the transport of MPs to the oceans (Li et al., 2020). However, it remains essential to assess the presence of MPs in other types of water bodies.

Despite the Water Framework Directive (WFD, 2000/60/EC) efforts to evaluate emerging compounds by redefining and updating the list of substances to be analysed every 2 years (Gomez Cortes et al., 2020; Gomez Cortes et al., 2022), MPs are not included in this watch list for assessing the ecological quality of reservoirs. These lentic ecosystems, formed by damming rivers, provide essential freshwater resources for drinking, irrigation, and recreation (INAG, 2006; Pinto et al., 2021a). However, the construction of dams disrupts the natural connectivity of rivers, leading to the accumulation of sediments, nutrients, and pollutants, including MPs, within the reservoirs (Di and Wang, 2018; Almeida et al., 2020; Pinto et al., 2021b). Moreover, reservoirs typically have longer water residence times and reduced turbulence compared to flowing rivers, further enhancing MPs accumulation (Sau et al., 2023; Queiroz et al., 2024). Given that reservoirs are essential water sources for human consumption, it is urgent to study their pollution levels from MPs and identify potential sources, highlighting the importance of including this evaluation in monitoring and management programs for these water bodies. While the topic is underexplored, studies, mainly from China, reveal concerning microplastic levels in reservoirs, such as the Three Gorges Reservoir, which recorded up to 12,611 MPs/m^3^ (Di and Wang, 2018). In Europe, research is limited and often relates to other topics such as sedimentation processes (Dhivert et al., 2022) or aquaculture (Bordós et al., 2019). In Portugal, Raposo et al. (2022) have already demonstrated the presence of MPs in the Alqueva reservoir, located in the south of the country; however, the study focused on their relationship with biofilms and other pollutants.

Therefore, this study aims to assess the occurrence of MPs in two Portuguese reservoirs, Rabagão and Aguieira, due to their distinct anthropogenic pressures and ecological characteristics. The research focuses on characterizing MPs in the surface water layer (photic layer) over 1 year and simultaneously characterizing the hydrographic basin and surrounding areas to identify potential sources of MPs pollution. Thus, this study aims to fill a gap in knowledge regarding the presence of MPs in freshwater reservoirs and emphasizes the urgent need to incorporate MPs into water quality monitoring programs to help mitigate their impacts.

## 2 Materials and methods

### 2.1 Study areas

**FIGURE 1:**
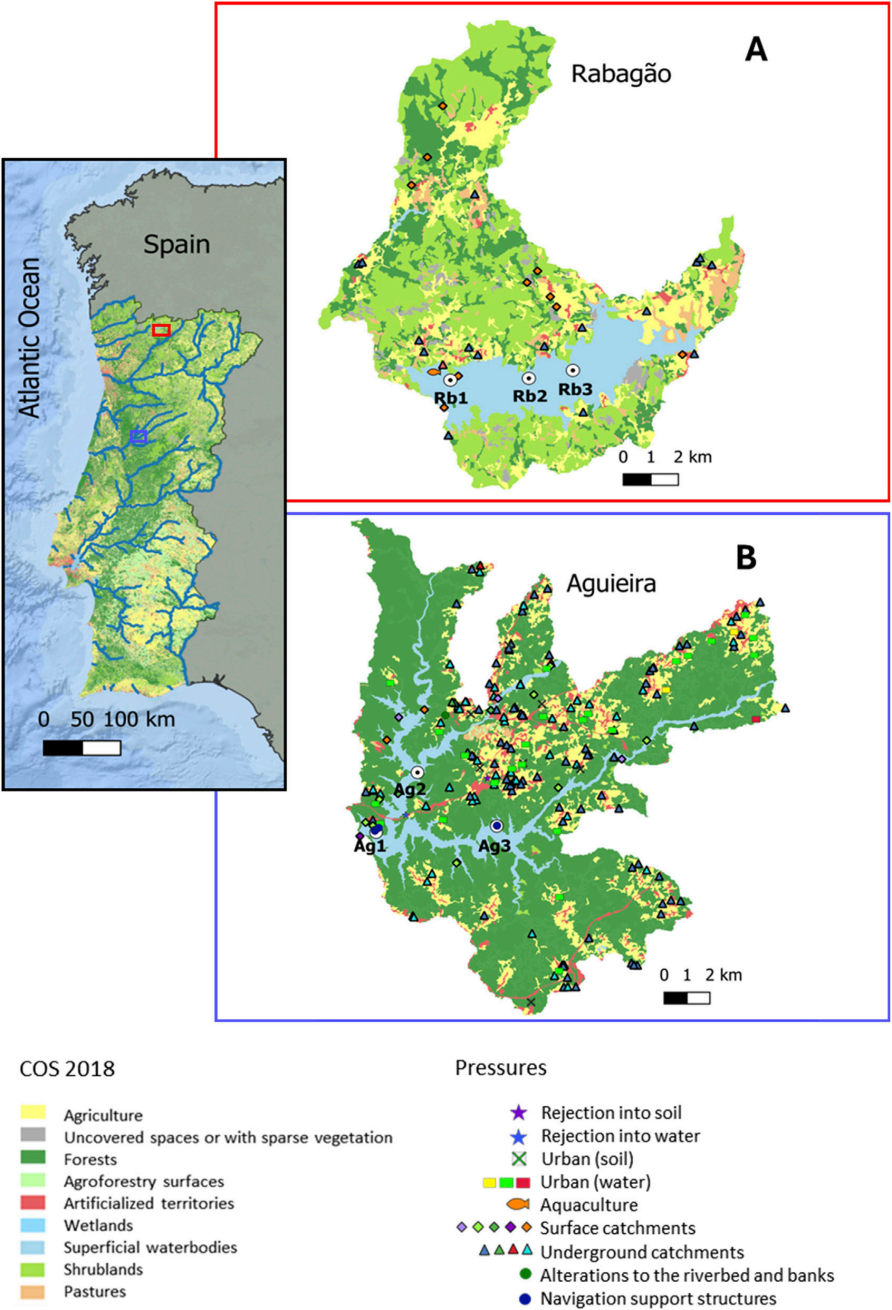
Map showing the location of the study areas corresponding to the Rabagão [**(A)**; in red] and the Aguieira [**(B)**; in blue] reservoirs, highlighting the land use within the hydrographic basin, based on the Land Use and Land Cover Map (COS) for 2018 V.2 Level 1. The pressures present in the hydrographic basins of the study areas, according to the 3^rd^ cycle of the River Basin Management Plans (PGRH; 2022–2027), are also presented. Additionally, the sampling sites used for this study are indicated within in each reservoir: Rabagão - Rb1 - 41°44′52.9″ N, 7°51′03.0″ W; Rb2 - 41°44′54.8″ N, 7°48′60.0″ W; Rb3 - 41°45′04.9″ N, 7°47′51.4″ W; and Aguieira - Ag1 - 40°20′32.7″ N, 8°11′18.4″ W; Ag2 - 40°21′59.2″ N, 8°10′01.3″ W; Ag3 - 40°20′41.6″ N, 8°07′34.0″ W.

To address the aims of this study, two Portuguese reservoirs classified as North type were selected (APA, 2023a; APA, 2023b): Rabagão and Aguieira (Figure 1). North type reservoirs correspond to water bodies located in mountainous areas, primarily used for hydroelectric power production. These have an average annual temperature below 15 °C and an average annual precipitation above 800 mm, siliceous substrate, a residence time generally less than 7 months, and water hardness below 50 µg CaCO_3_/L (INAG et al., 2010; APA, 2021).

Rabagão reservoir (also known as Pisões reservoir: Figure 1A), located in Montalegre, is part of the Cávado river basin and is primarily fed by the Rabagão river, with additional water diverted from the Alto Cávado reservoir (POAAR, 2009). Rabagão dam, completed in 1964, was originally constructed for hydroelectric power generation but is now also used for water storage and recreational activities (Almeida et al., 2020). The reservoir has a total capacity of 568,700 dam^3^ and a basin area of 78.03 km^2^ (APA, 2023b). The region has a temperate-Mediterranean climate with continental influences (APA, 2016a). Regarding land use and land cover in the region, forests are predominant, followed by agricultural and agro-forestry areas (APA, 2012a; Figure 1A). This reservoir has consistently maintained a Good Ecological Potential through the WFD planning cycles (APA, 2023b).

Aguieira reservoir (Figure 1B), located in Coimbra, is part of the Mondego River basin and receives water from the Mondego, Dão, and Criz rivers (Pinto et al., 2021a). The dam was constructed in 1981 for energy production and public supply (POAA, 2005); however, the reservoir is currently also utilized for recreational activities, often attracting a high number of people to river beach areas, particularly in the summer. The reservoir has a total capacity of 423,000 dam^3^ and a basin area of 208.35 km^2^ (POAA, 2005). The region has a temperate-Mediterranean climate, and the surrounding land is mainly used for forestry, agriculture, and industrial activities (APA, 2012b; Pinto et al., 2021a; Figure 1B). Aguieira Ecological Potential classification has fluctuated, achieving Moderate in the 1^st^ and 3^rd^ WFD cycles, but Poor in the 2^nd^, failing to meet the WFD’s ecological objectives (APA, 2023c) Moreover, Aguieira reservoir was included in the WFD inter-calibration study for this water body typology (Pinto et al., 2025).

### 2.2 Characterization of the hydrographic basins of the study areas

Using QGIS v.3.28.13, the hydrographic basins of the Rabagão and Aguieira reservoirs were delineated, including surrounding areas that drain into the reservoirs. Rabagão basin also incorporates the Alto Cávado reservoir. Land use and cover were characterized using 2018 v.2 Land Use and Land Cover Map (COS, 2018), focusing on level 1 categories represented by different colours in Figures 1A,B: artificialized territories (1), agriculture (2), pastures (3), agroforestry surfaces (4), forests (5), shrublands (6), uncovered spaces or with sparse vegetation (7), and surface water bodies (9).

Additionally, to identify potential sources of MPs, the presence of pressures in the study areas was analysed: punctual qualitative pressures (e.g., industry, tourism, waste, urban, aquaculture), punctual quantitative pressures (e.g., surface catchments), and hydromorphological pressures (e.g., alterations to the riverbed and banks, navigation support structure) as identified in the 3^rd^ planning cycle of the River Basin Management Plans - PGRH (2022–2027).

### 2.3 *In situ* procedures

Throughout 2023, six sampling periods were defined: three in the summer with a minimum interval of 3 weeks, according to the WFD monitoring program for reservoirs of Northern type (APA, 2021), and additional sampling campaigns in the other seasons (winter, spring, and autumn). The WFD monitoring program recommends conducting three summer samplings for Northern-type reservoirs, as this is considered an ecologically more critical period. During this time, higher temperatures, increased solar radiation (which promotes the growth of phytoplankton and cyanobacteria), lower water renewal, and longer residence times are typically observed in reservoirs, along with a potential increase in anthropogenic pressures (e.g., tourism, irrigation). Additionally, reservoirs tend to exhibit greater thermal stability and vertical stratification during summer, factors that significantly influence the distribution of oxygen, nutrients, and aquatic organisms. The distribution of the remaining sampling periods was designed to cover all seasons (allowing for the discrimination of seasonal variations) and to encompass the different pressures that may exist throughout the year. Also, sampling in each reservoir was carried out on the same day and at similar times in the morning between reservoirs. In each reservoir, three sampling sites were defined in the area of lentic ecosystem (Figures 1A,B), considering the size of the reservoir, the heterogeneity of the water body (including proximity to different anthropogenic pressures and surrounding land uses), and, whenever possible, their integration into the national monitoring program (SNIRH, 2025; Pinto et al., 2025). The selected sites included areas near the dam as well as more central zones, also accounting for available resources, logistical limitations, and accessibility.

In the same day (for each reservoir) a sub-surface water sample (<0.5 m depth; three replicates) was collected at each site, using a glass bottle of 1 L for subsequent MPs analysis. This methodology and water volume for replicates have already demonstrated effectiveness, successfully capturing even the smallest MPs (e.g., Prata et al., 2020; Prata et al., 2024). *In situ*, several general physical and chemical parameters were measured, to determine the Ecological Potential according to the WFD approach: pH, conductivity (μS/cm), temperature (°C), and dissolved oxygen (mg/L and %), using a multiparameter probe (Multi 3630 IDS SET F). Additionally, transparency was measured using a Secchi disk, and a sub-surface water sample (<0.5 m depth) was also collected for further laboratory analysis. For logistical reasons, the two reservoirs were sampled in consecutive days.

### 2.4 Laboratory procedures

#### 2.4.1 Physical, chemical, and biological parameters

In the laboratory, several physical, chemical, and biological parameters were assessed to determine the Ecological Potential of the studied reservoirs in accordance with the WFD approach. Biochemical oxygen demand (BOD_5_, mg/L) was calculated according to APHA (1989). Additionally, the samples were filtered in triplicate using a Whatman GF/C filter (47 mm diameter and 1.2 μm pore) to determine the total suspended solids (TSS, mg/L), following the methodology described in APHA (1989), and for the quantification of chlorophyll *a* concentration (biological element regarding WFD metrics) according to the method described by Lorenzen (1967). Furthermore, the concentrations of orthophosphate (mg PO_4_/L), ammonia nitrogen (mg NH_4_/L), nitrate (mg NO_3_/L), and nitrite (mg NO_2_/L) were quantified using a Skalar Sanplus Segmented Flow Autoanalyzer, according to Skalar methods: M461-318 (EPA 353.2), M155-008R (EPA 350.1), and M503-555R (Standard Method 450-P).

#### 2.4.2 Microplastics analysis

For the analysis of MPs, the methodology described by Gago et al. (2018) and Frias et al. (2018) was followed. Initially, water samples from both reservoirs were pre-filtered using a 1 mm mesh to remove coarse organic material such as leaves and larger zooplankton. Due to the minimal organic content, digestion was not necessary, as outlined by Gago et al. (2018) and Frias et al. (2018). The water samples were vacuum filtered using Whatman GF/C glass microfiber filters (47 mm diameter, 1.2 µm pore size), and the filters were dried in an oven at 40 °C until a constant weight was achieved, then sealed in Petri dishes for later observation with a Leica MZ7.5 binocular stereoscope.

The evaluation of MPs in each sample was conducted considering: the type (fibre, fragment, paint, film, or pellet) and colour (black, blue, red, multicoloured, transparent, among others) (Karami et al., 2017; Gago et al., 2018). Additionally, all particles were measured according to type (fibres: length measurement; other types: height and width measurements) using the Leica Application Suite (LAS) software. For the chemical analysis of MPs, approximately 10% of the total particles were characterized, following the recommendations of Hanke et al. (2014) and Cowger et al. (2024), using an Attenuated Total Reflectance - Fourier Transform Infrared Spectroscopy (ATR-FTIR), with a Spectrum Two™ spectrometer (PerkinElmer, United States of America) equipped with a single-reflection diamond crystal accessory. Only particles larger than 0.3 mm were considered for the chemical characterization, due to equipment limitations (Liu et al., 2024). The particles were randomly selected using a stratified approach by type and color, ensuring representation across different morphotypes. The spectrum was acquired in the range of 4,000 to 400 cm^−1^, with a resolution of 4 cm^−1^ and an accumulation of 64 scans, with automatic CO_2_/H_2_O atmospheric correction. Between each measurement, the ATR diamond crystal was cleaned with 70% isopropanol, and a new background measurement was performed every four measurements. The correspondence between the obtained spectrum and the polymer type was determined by comparison with reference spectra available in the instrument’s software library (Spectrum 10, PerkinElmer). Spectra with a quality match index above 60% were accepted (Hanke et al., 2014), with additional comparison with the literature.

The contamination from all potential MPs sources, including laboratory clothing, surfaces, and air, was carefully controlled. All laboratory procedures were performed using glassware, and Petri dishes were only opened when strictly necessary to minimize airborne contamination. Also, air blank samples were placed in open petri dishes, near the filtration system, and subjected to the same drying process to assess possible airborne contamination. The number of particles detected was very low (<10 particles), indicating negligible contamination and validating the results obtained (Liu et al., 2019; Shruti and Kutralam-Muniasamy, 2023).

### 2.5 Statistical analysis

The results of physical, chemical, and biological parameters of summer samples were used to classify the Ecological Potential of the studied reservoirs according to the reference values from the WFD for Northern type reservoirs (APA, 2023a). The Ecological Potential of each sample was determined based on these classifications, adhering to the “one out-all out” principle (WFD, 2000/60/EC).

Regarding the analysis of MPs, a one-way ANOVA followed by a Tukey test (using SigmaPlot v.11.0) was conducted to determine differences in the concentration of MPs among sampling sites within each sampling period. The same analysis was conducted to determine differences in concentration of MPs between sampling periods for each reservoir. Additionally, based on the MPs content in the two reservoirs and all the parameters that allowed the characterization of the study areas (COS, anthropic pressures, and WFD parameters), a dendrogram was constructed using the complete linkage method, generated based on Euclidean distances, using Primer v7.0.11 software to understand the similarity among the sampling sites.

## 3 Results

### 3.1 Characterization of the hydrographic basins of the study areas

Table 1 presents the characterization of the hydrographic basins of the study areas, Rabagão and Aguieira, based on the level 1 classification from 2018 v.2 COS. Regarding the Rabagão reservoir, shrubland are the predominate area (39.5%), followed by forests (19.6%). Agriculture (15.7%) also accounted for a significant percentage of land use and land cover in this study area. Additionally, though less representative, areas of pastures, uncovered spaces or with sparse vegetation, artificialized territories, and agroforestry surfaces were also observed (5.15%, 2.83%, 1.43%, and 0.012%, respectively; Table 1). On the other hand, the area corresponding to the Aguieira reservoir is predominantly covered by forests (69.8%), also showing a percentage of land dedicated to agriculture (15.2%), similar to the Rabagão reservoir. Furthermore, this hydrographic basin also presents areas of artificialized territories, pastures, shrublands, and agroforestry surfaces (5.52%, 0.555%, 0.415%, and 0.098%, respectively; Table 1).

**TABLE 1:** Land use and cover characterization (%) of the hydrographic basins of Rabagão and Aguieira reservoirs, based on the level 1 classification from the Land Use and Land Cover Map (COS) for 2018, version 2. The pressures identified in the hydrographic basins of Rabagão and Aguieira reservoirs, according to the 3^rd^ cycle of the River Basin Management Plans (2022-2027), are also presented.

COS 2018 v.2 level 1		Rabagão	Aguieira
		%	%
1	Artificialized territories	1.43	5.52
2	Agriculture	15.7	15.2
3	Pastures	5.15	0.555
4	Agroforestry surfaces	0.012	0.098
5	Forests	19.6	69.8
6	Shrublands	39.5	0.415
7	Uncovered spaces or with sparse vegetation	2.83	0
9	Superficial waterbodies	15.8	8.38
Pressures		Total = 28	Total = 194
Rejection into soil		0	1
Rejection into water		0	1
Urban (soil)	Secondary	0	7
Urban (water)	Primary	0	2
	Secondary	0	24
	Unknown	0	1
Aquaculture		1	0
Surface catchments	Public supply	0	3
	Agriculture	0	9
	Green spaces	0	2
	Hydroelectric	0	1
	Unknown	10	2
Underground catchments	Agriculture	17	80
	Green spaces	0	2
	Livestock	0	1
	Unknown	0	54
Alterations to the riverbed and banks		0	1
Navigation support structures		0	3

The pressures present in the hydrographic basins of the study areas, according to the 3^rd^ cycle of the River Basin Management Plans (PGRH; 2022–2027) showed that Rabagão presents punctual quantitative pressures, including underground catchments for use in agriculture as well as surface catchments (Table 1). An aquaculture plant, specifically trout farming, is the only recorded punctual qualitative pressure in this reservoir (Table 1). The hydrographic basin of the Aguieira reservoir presents a higher number of recorded pressures, with a particularly high number of underground catchments (n = 137), mainly for agricultural purposes. Surface catchments are also noted, for agriculture, public water supply, and hydroelectric power generation (Table 1). Regarding punctual qualitative pressures, those resulting from the urban sector stand out, particularly discharges from WWTPs into water bodies and soil. Hydromorphological pressures were also recorded, including the presence of navigation support structures near the sampling sites in this reservoir, as well as alterations to the riverbed and banks (Table 1).

### 3.2 Water quality assessment of the study areas

Table 2 presents the results of physical, chemical, and biological parameters determined for each water sample from each reservoir, as well as the reference values indicated by the Water Framework Directive (APA, 2023a) for the assessment of Ecological Potential. It also includes the final classification of the Ecological Potential of each summer sample (Sum1, Sum2, and Sum3), based on the worst rating obtained.

**TABLE 2:** Results of physical-chemical, and biological parameters, and Ecological Potential, according to the reference values defined by the WFD for reservoirs (APA, 2023a), related to the study areas of Rabagão and Aguieira (sampling sites: Rb1, Rb2, and Rb3, Ag1, Ag2 and Ag3, respectively; sampling periods: winter (Win), Spring (Spr), Summer 1 (Sum1), Summer 2 (Sum2), Summer 3 (Sum3), and Autumn (Aut)).

Seasons	Sites	Conductivity (μS/cm)*	Temperature (°C)*	Secchi disk (m)*	pH	Dissolved oxygen (mg O_2_/L)	Dissolved oxygen (%)	TSS (mg/L)*	BOD_5_ (mg/L)	Orthophosphate (mg PO_4_/L)	Ammonia nitrogen (mg NH_4_/L)	Nitrate (mg NO_3_/L)	Nitrite (mg NO_2_/L)	Physical/Chemical classification	Clorophyll *a* (mg/L)	Biological classification	Final classification
Excellent - good					6.5–8.5	8.0–12.0	80–115	12.5	3.0	0.08	0.10	2.0	0.010				
Good - moderate		100	6.5–25.5	2.3	6.0–9.0	6.0	70–125	25	4.0	0.12	0.20	3.0	0.020		7.9		
Win	Rb1	20.4	8.60	3.3	6.920	10.8	102.8	12.01	0.96	0.069	0.005	0.650	<0.0115		6.09		
	Rb2	20.5	8.00	3.5	7.060	10.8	101.4	12.63	1.4	0.066	0.0045	0.639	<0.0115		6.28		
	Rb3	25.4	8.00	3.1	7.191	10.6	100.3	13.16	1.3	0.066	0.005	0.634	<0.0115		6.01		
Spr	Rb1	24.4	18.8	3.0	7.776	9.05	107.1	14.01	0.97	0.089	0.004	0.285	<0.0115		6.19		
	Rb2	39.3	17.7	3.0	7.475	9.21	106.5	12.19	0.65	0.090	0.004	0.298	<0.0115		5.16		
	Rb3	19.4	16.8	3.0	7.372	9.56	108.9	11.53	0.56	0.091	0.005	0.317	<0.0115		3.72		
Sum1	Rb1	23.4	21.6	3.0	7.859	8.18	102.7	15.15	0.15	0.060	0.023	0.040	<0.0115	Good	3.76	Good	Good
	Rb2	20.9	20.9	3.0	7.760	8.13	101.9	14.98	0.75	0.070	0.029	<0.016	<0.0115	Good	3.21	Good	Good
	Rb3	39.7	21.9	3.0	8.060	8.15	103.3	14.85	0.80	0.050	0.022	<0.016	<0.0115	Good	3.34	Good	Good
Sum2	Rb1	27.1	25.9	3.0	7.767	7.80	106.0	15.08	1.2	0.040	0.038	<0.016	<0.0115	Good	2.20	Good	Good
	Rb2	19.6	24.9	3.0	7.560	8.08	107.6	15.13	1.6	0.030	0.024	<0.016	<0.0115	Good	1.66	Good	Good
	Rb3	22.8	25.0	3.0	7.787	8.04	107.5	14.71	1.1	0.040	0.026	<0.016	<0.0115	Good	2.12	Good	Good
Sum3	Rb1	23.5	19.5	3.0	7.831	8.55	102.4	17.18	0.48	0.030	0.022	<0.016	<0.0115	Good	6.59	Good	Good
	Rb2	19.7	19.2	3.0	7.460	8.66	102.5	12.62	1.6	0.030	0.043	<0.016	<0.0115	Good	5.82	Good	Good
	Rb3	19.6	19.4	3.0	7.109	8.58	103.2	11.85	1.2	0.030	0.021	<0.016	<0.0115	Good	5.73	Good	Good
Aut	Rb1	24.9	11.6	2.5	7.508	9.45	94.60	24.35	1.0	<0.024	0.022	0.539	0.0710		10.5		
	Rb2	22.3	11.4	2.5	7.285	9.39	94.80	18.60	0.91	<0.024	0.018	0.460	0.0710		9.97		
	Rb3	21.8	11.3	2.5	7.117	9.47	95.50	19.04	1.3	<0.024	0.013	0.430	0.0710		10.2		
Win	Ag1	60.1	12.9	2.0	8.780	12.3	117.4	16.88	1.9	0.456	0.0036	2.37	<0.0115		14.0		
	Ag2	85.7	17.4	2.0	8.090	10.9	114.0	16.65	0.82	0.544	0.0044	2.78	0.0450		7.85		
	Ag3	57.7	14.8	1.5	9.000	12.6	123.1	17.68	1.3	0.465	0.0036	1.72	<0.0115		19.6		
Spr	Ag1	75.2	19.8	3.3	9.329	9.38	103.8	14.42	1.7	0.098	0.0020	0.790	<0.0115		7.40		
	Ag2	79.6	20.8	3.4	9.773	9.86	111.5	15.48	1.2	0.100	0.0009	1.28	<0.0115		13.4		
	Ag3	71.5	20.2	3.4	9.403	9.36	104.4	15.65	1.4	0.093	0.0020	0.330	<0.0115		7.89		
Sum1	Ag1	81.5	25.2	2.3	10.14	9.26	114.7	22.08	1.9	0.240	0.0039	0.110	<0.0115	Moderate	13.0	Moderate	Moderate
	Ag2	109	26.2	1.9	10.28	9.37	117.7	16.03	1.5	0.280	0.0043	0.020	<0.0115	Moderate	14.8	Moderate	Moderate
	Ag3	83.5	26.0	1.6	9.910	9.01	112.1	16.75	1.0	0.100	0.0041	0.040	<0.0115	Moderate	12.7	Moderate	Moderate
Sum2	Ag1	78.1	25.8	2.1	9.551	8.65	107.1	15.65	0.90	0.130	0.0100	<0.016	<0.0115	Moderate	4.15	Good	Moderate
	Ag2	102	27.3	2.3	9.734	8.62	110.0	15.82	2.2	0.210	0.0134	0.020	<0.0115	Moderate	3.68	Good	Moderate
	Ag3	74.2	26.3	2.8	8.564	8.52	106.8	13.95	1.2	0.050	0.0229	<0.016	<0.0115	Good	2.41	Good	Good
Sum3	Ag1	78.5	20.9	2.0	7.699	7.71	87.00	15.55	0.91	0.240	0.0302	0.180	<0.0115	Moderate	17.2	Moderate	Moderate
	Ag2	88.1	22.3	2.0	7.761	8.31	96.50	15.58	0.96	0.190	0.0290	<0.016	<0.0115	Moderate	21.8	Moderate	Moderate
	Ag3	77.0	21.5	2.3	7.486	8.20	94.00	13.85	0.60	0.190	0.0297	0.330	<0.0115	Moderate	5.09	Good	Moderate
Aut	Ag1	67.0	16.5	3.1	6.992	8.28	86.50	13.02	0.45	0.139	0.0100	2.29	0.0690		2.95		
	Ag2	70.6	16.8	3.0	6.842	8.06	84.70	12.72	0.29	0.140	0.0319	3.15	0.0680		2.16		
	Ag3	60.2	16.3	3.0	6.875	8.26	85.60	14.45	0.61	0.174	0.0180	2.26	0.0700		1.57		

The colors blue, green, and yellow represent Excellent, Good, and Moderate Ecological Potential, respectively. *Parameters integrated complementarily, considered penalizing only when quality below Good is also verified for another of the parameters presented.

Regarding summer samples of the Rabagão reservoir (Table 2), physical, chemical, and biological parameters, were classified as Good, resulting in a final classification of Good Ecological Potential. The physical and chemical classification of the summer samples was mainly influenced by NO_2_ concentrations, which were always above the maximum defined for the Excellent classification. It was also observed a value of O_2_ (mg/L) below the Excellent threshold, coinciding with the highest temperature recorded, in Rb1 in Sum2. Moreover, in almost all cases TSS values exceeded the threshold for Excellent, although it did not impact the final classification. In all summer samplings, chlorophyll *a* concentration stands between the Good classification, with Rb2 in Sum2 recording the lowest concentration (1.66 mg/L). Considering the remaining sampling periods, higher concentrations of NO_2_ and chlorophyll *a* were observed in Aut, which may negatively affect the quality of this reservoir in this period.

Regarding the Aguieira reservoir (Table 2), the results of physical and chemical parameters allow classifying this reservoir as Moderate, except for Ag3 in Sum2, which was classified as Good. The lower classification was mainly due to high pH values, particularly in the Sum1 and Sum2 samples, and high PO_4_^3-^ concentrations, especially in Ag1 and Ag2. High conductivity and temperature values, as well as transparency levels below the Good threshold, were observed, which were classified as Moderate, though they only served as penalizing factors. Regarding the biological classification, chlorophyll *a* concentration for all sampling sites in Sum2 and Ag3 in Sum3 fell within the limits for a Good classification, but in the remaining periods, the values were high, resulting in a Moderate classification. Considering the other sampling periods, it was observed high concentrations of chlorophyll *a* in Win and Spr, as well as of PO_4_ and NO_2_ in Win and Aut and NO_3_ in Aut. It was also observed high pH values in Spr. Additionally, Win presented high O_2_ levels and low transparency values. All these factors also contribute to the loss of quality of this reservoir.

### 3.3 Microplastic analysis

**FIGURE 2:**
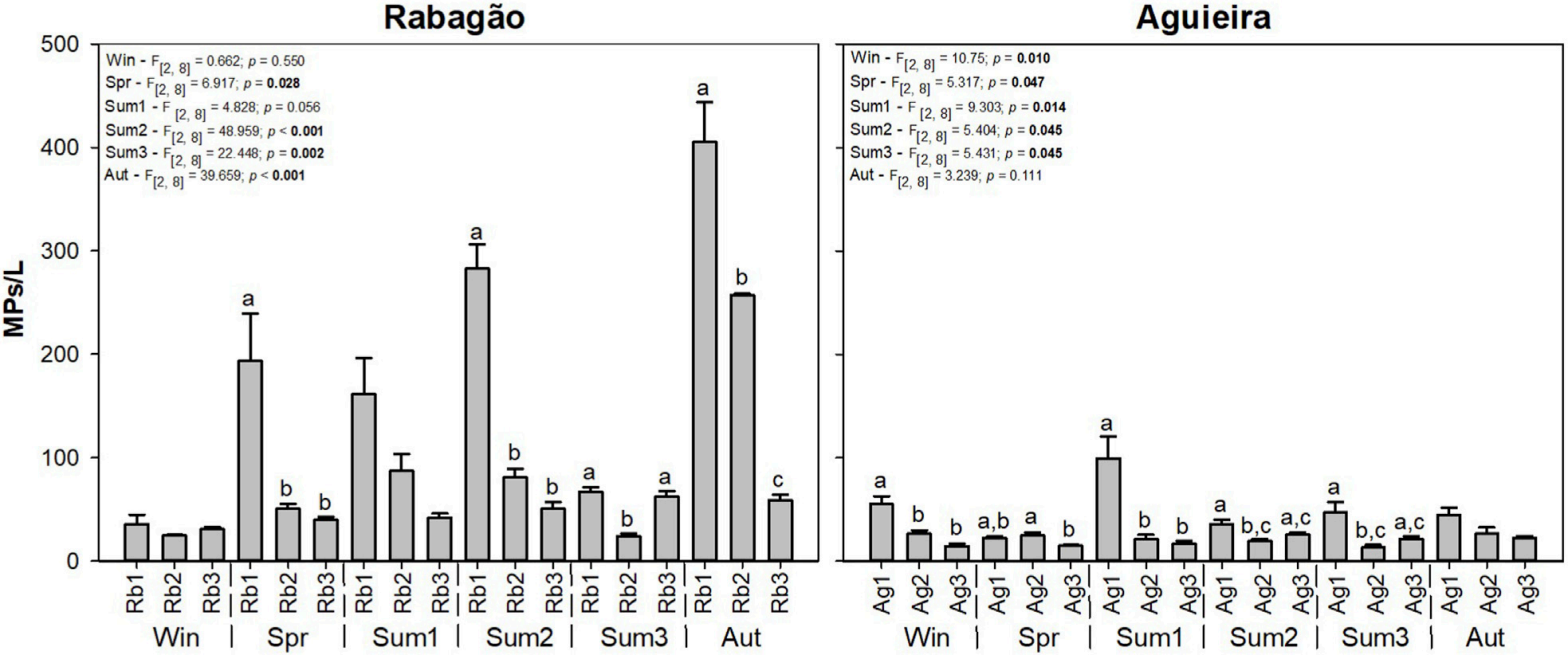
Results of microplastic concentration (MPs/L) in each sampling period (Win - winter, Spr - spring, Sum1 - summer 1, Sum2 - summer 2, Sum3 - summer 3, and Aut - autumn) and sampling site, in the Rabagão (Rb1, Rb2, and Rb3) and Aguieira (Ag1, Ag2, and Ag3) reservoirs. Different letters (a, b, c) represent significant differences between sampling sites in each sampling period. Degrees of freedom, F-statistic, and *p*-value are presented for each sampling period considered. Significant values are highlighted in bold (*p* < 0.05).

A total abundance of 5,862 MPs was identified in the Rabagão reservoir and 1,658 MPs in the Aguieira reservoir. MPs were found at all sampling sites and all sampling periods in both reservoirs, and a significant increase of MPs concentration was observed in the sites closest to the dams (Figure 2). In Rabagão reservoir, no significant differences were observed in MPs concentrations between sampling sites in the sampling campaigns of Win and Sum1 (Figure 2). In Spr and Sum2 a significant increase was observed in Rb1 and no significant differences were observed between Rb2 and Rb3. In Sum3, Rb2 stood out with the significantly lowest value (Figure 2). In Aut, significant differences were observed between all sites, with site Rb1 having the highest MPs concentration (405 ± 39 MPs/L) and Rb3 the lowest (Figure 2). Regarding the Aguieira reservoir (Figure 2), the MPs concentration was apparently lower, compared to the Rabagão reservoir. The high MPs concentration was observed at Ag1 in Sum1 (100 ± 21 MPs/L), and the lowest was observed at Ag2 in Sum3 (13 ± 2). No significant differences were observed between sampling sites in Aut (Figure 2). A significant decrease in MPs was observed in Ag2 and Ag3 at Win and Sum1. In Spr, a different pattern was observed with the lowest MPs concentrations detected at Ag1 and Ag3. In Sum2 and Sum3, the trend was similar, with the highest MPs concentration observed at Ag1 followed by Ag2, but not significantly different from Ag3. For both reservoirs, no significant differences were observed between MPs concentrations across sampling periods (Rabagão - F_[5,17]_ = 1.752 *p* = 0.198; Aguieira - F_[5,17]_ = 0.401 *p* = 0.839).

**FIGURE 3:**
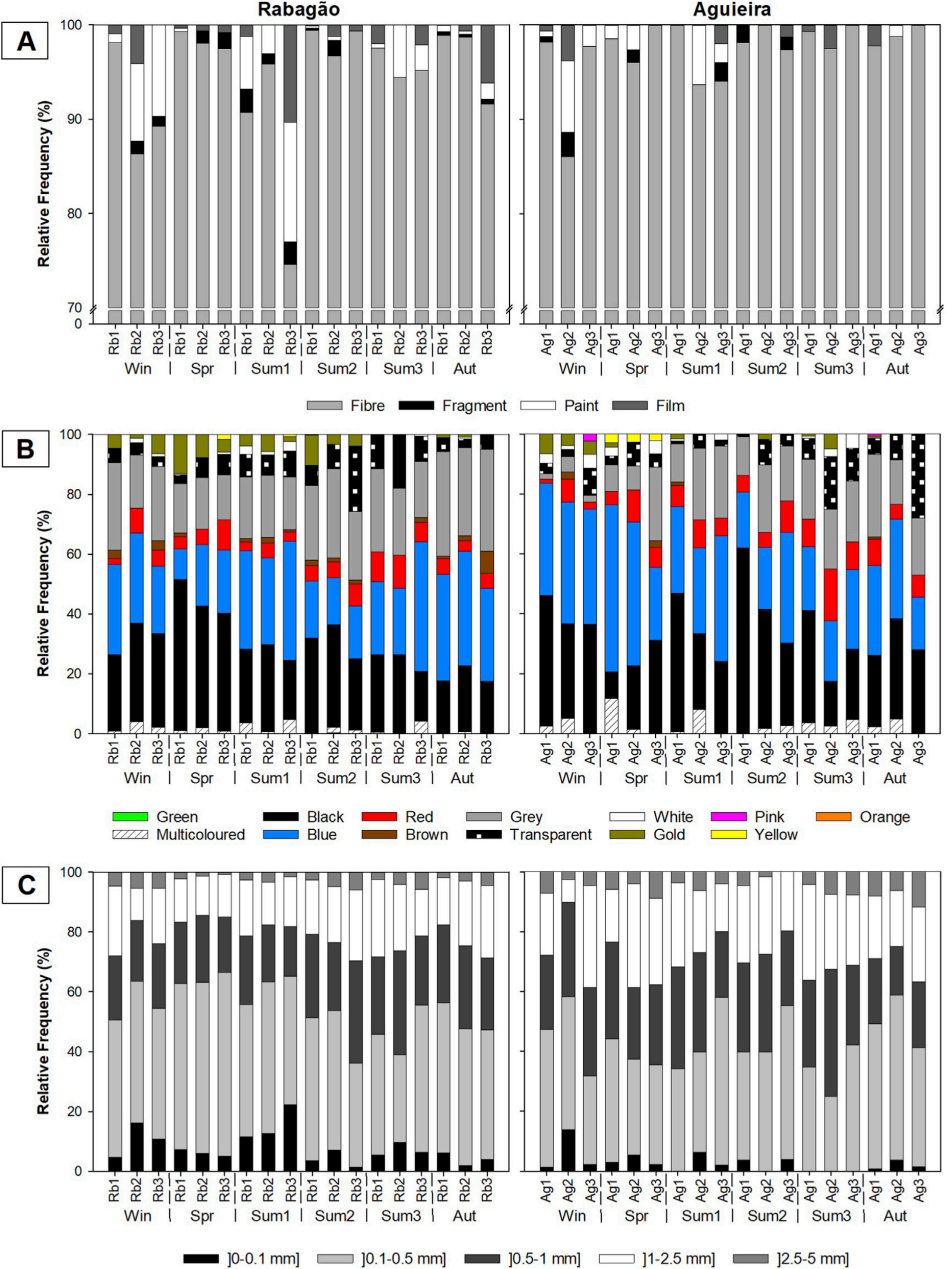
Results of microplastics characterization (%) based on: **(A)** type; **(B)** colour; and **(C)** size category, for each sampling period (Win - winter, Spr - spring, Sum1 - summer 1, Sum2 - summer 2, Sum3 - summer 3, and Aut - autumn) and sampling site, in the Rabagão (Rb1, Rb2, and Rb3) and Aguieira (Ag1, Ag2, and Ag3) reservoirs.

Regarding the type of MPs, pellets and foams were not observed. The other four types of MPs commonly described in the literature (fibre, fragment, film, paint) were found in both reservoirs, with fibres being the predominant type, ranging from 74.6% to 99.4% in the Rabagão reservoir and from 86.1% to 100% in the Aguieira reservoir (Figure 3A). Although at a much lower percentage, paint particles represent the second most identified type of microplastic in both reservoirs, ranging up to 12.7% in the Rabagão and up to 7.6% in the Aguieira reservoir. Films and fragments were the least commonly observed types of MPs, with their highest percentages recorded at 10.3% and 2.5%, respectively, in the Rabagão reservoir, and 3.8% and 2.5%, respectively, in the Aguieira reservoir (Figure 3A).

A total of 13 different colours were identified among the observed MPs (Figure 3B). MPs with faded colours (partially transparent and partially of another colour) were also counted as multicoloured. In general, in the Rabagão reservoir, the predominant colours were blue (ranging from 10.2% to 43.3%), black (ranging from 16.6% to 50.5%), and grey (ranging from 15.1% to 35.0%). No pink MPs were identified in this reservoir, and the least identified colours were yellow, green, and orange (with maximum percentages of 1.7%, 0.4%, and 0.1%, respectively; Figure 3B). The same pattern of predominant colours was observed in the Aguieira reservoir (blue between 17.6% and 55.9%, black between 8.8% and 62.0%, and grey between 1.8% and 27.6%), with green and orange being the only colours not identified in this reservoir. Brown, pink, and yellow were the least observed colours among the MPs identified in this reservoir (with the maximum percentages of 2.5%, 2.3%, and 2.9%, respectively; Figure 3B).

The distribution of the identified MPs by different size categories is represented in Figure 3C. Regarding the Rabagão reservoir, the most representative size category of MPs was between 0.1 and 0.5 mm for all sampling sites and periods. This size category was also predominant in most sites and sampling periods in the Aguieira reservoir, except in Ag3 during Win and Ag2 during Spr and Sum3, where larger MPs prevailed, specifically in the 0.5–1 mm and 1–2.5 mm categories. Considering the overall samples from both reservoirs, the smallest (0–0.1 mm) and largest (2.5–5 mm) MPs were the least observed.

**FIGURE 4:**
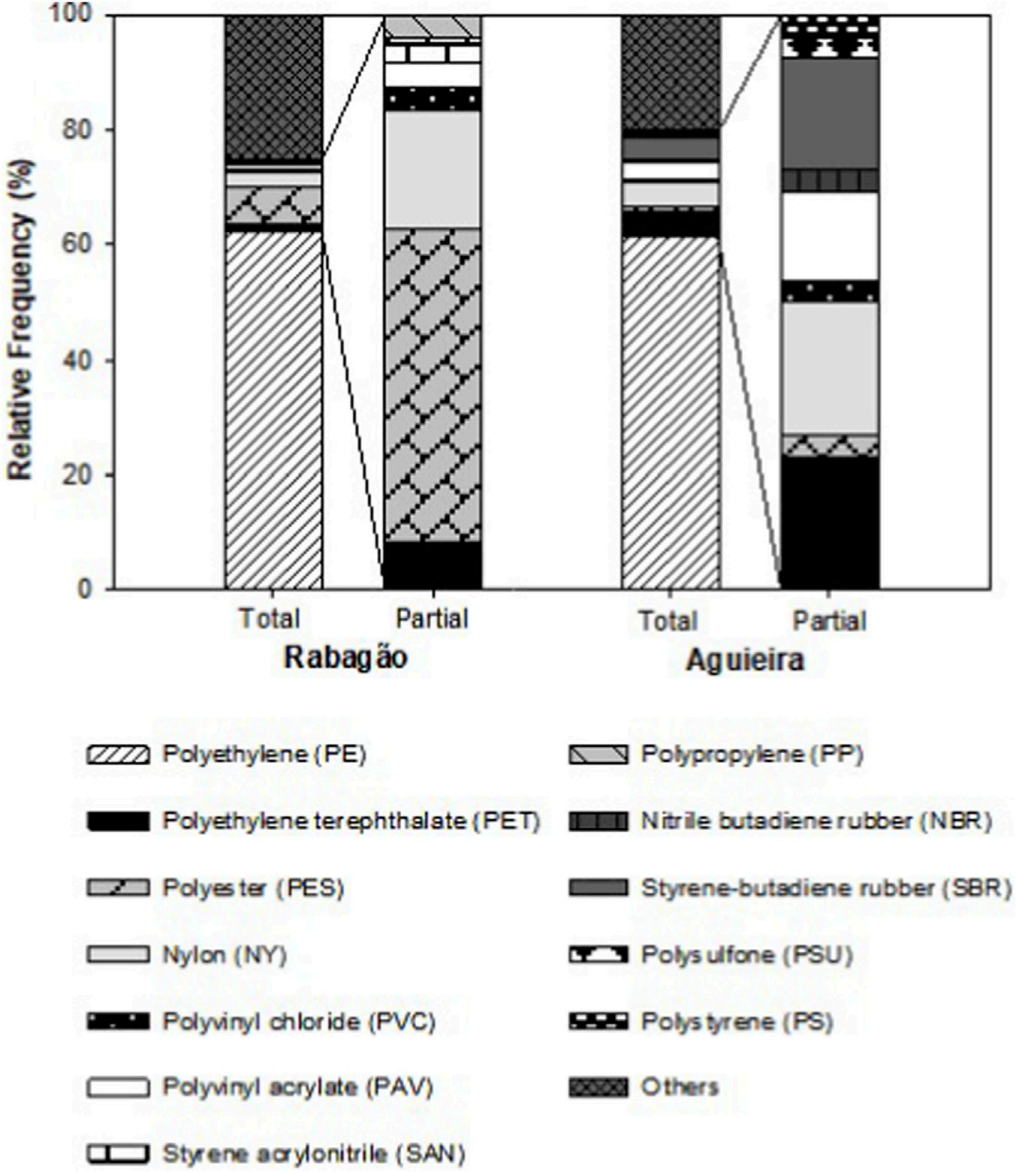
Polymer types (%) identified through ATR-FTIR analysis of microplastic particles found in the Rabagão and Aguieira water samples.

**FIGURE 5:**
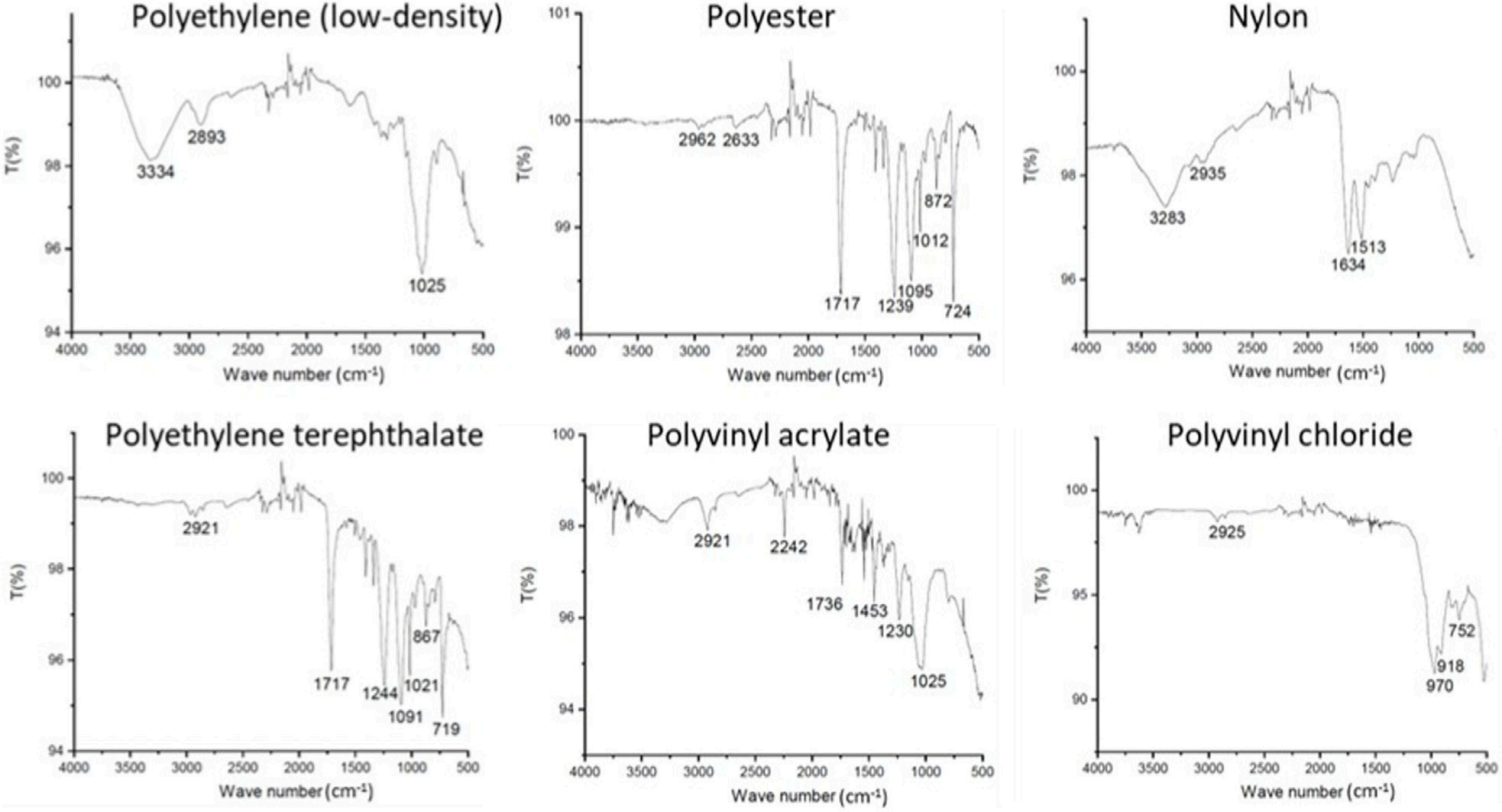
Examples of ATR-FTIR spectra of the most frequent microplastic samples found in the studied reservoirs, showing the predominant polymers, where T represents transmittance (%).

The ATR-FTIR analysis of the MPs particles analysed revealed that polyethylene (PE (low-density); Figures 4, 5) was the predominant polymer found in both reservoirs under study (≈60% of the MPs). However, other polymers such as polyethylene terephthalate (PET), polyester (PES), nylon (NY), polyvinyl acrylate (PAV), and polyvinyl chloride (PVC) were also commonly identified in both reservoirs (Figures 4, 5). Among the particles analysed from the Rabagão reservoir, 62.4% were PE, while PES accounted for 6.7%, NY represented 2.6%, PET only 1%, and both PVC and PAV made up less than 1% each. Styrene acrylonitrile (SAN) and polypropylene (PP) were also identified for this reservoir, representing less than 1% each (Figure 4). In the case of the Aguieira reservoir, PE constituted 61.4% of the particles analysed (10%). PET and NY each accounted for 4.3% of the analysed samples, while PAV constituted 2.9%, and both PES and PVC were below 1%. Nitrile butadiene rubber (NBR), polysulfone (PSU), and polystyrene (PS) polymers were also identified for this reservoir, each accounting for less than 1%, in addition to styrene-butadiene rubber (SBR), which represented 3.6% (Figure 4).

**FIGURE 6:**
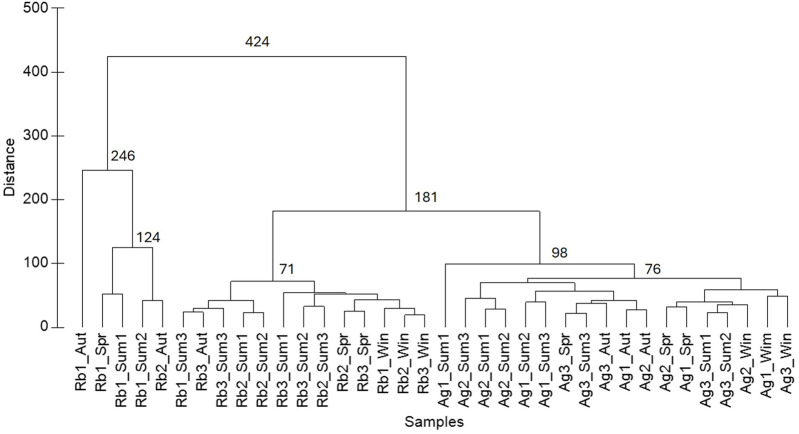
Dendrogram related to the analysis of microplastics (type, colour, size category, and FTIR analysis), and the characteristics of the study areas (COS and pressures (Table 1), and WFD parameters (Table 2)). Sampling sites: Rb1, Rb2, Rb3 (Rabagão), Ag1, Ag2, Ag3 (Aguieira); and sampling periods: winter (Win), Spring (Spr), Summer 1 (Sum1), Summer 2 (Sum2), Summer 3 (Sum3), and Autumn (Aut).

Figure 6 shows the combined analysis of MPs (type, colour, size category, and FTIR analysis) observed across all samples and the characterization of the study areas (COS, anthropic pressures, and WFD parameters). The dendrogram reveals two main groups. The first group (distance = 424) separates the Rb1 samples from different sampling periods together with Rb2_Aut from the remaining Rabagão sites and all Aguieira samples. Within this first group, Rb1_Aut stands out from the other samples of the same group (distance = 246). The second group is subdivided into two subgroups (distance = 181): one including the remaining Rabagão sampling sites and the other comprising the Aguieira samples. Within the Rabagão subgroup, a similarity between the summer samples from Rb2 and Rb3 was observed. The Aguieira cluster is further subdivided into smaller groups, with Ag1_Sum1 being the most distinct site (distance = 98). This hierarchical structure highlights a clear separation between the two reservoirs and among the different sites in the Rabagão reservoir. Given that COS and anthropic pressures were considered uniform across all sampling points within each reservoir, the MPs content, along with physical, chemical, and biological parameters, had the greatest influence on the formation of the identified groups. It can be observed that, except for the first group, which consists mainly of Rb1, samples from the same season/sampling period tend to cluster together. This pattern can be explained by the influence of the analysed parameters, that which likewise tend to vary seasonally. Additionally, since all sampling sites generally exhibited a great diversity of MPs, the grouping of locations is primarily due to MPs concentration rather than the analysed characteristics (type, colour, size and FTIR analysis). This can explain the distance observed between Ag1 and Rb1, and other sampling sites within each reservoir in almost all cases, as an increase in the concentration of MPs was observed from upstream to the dam in each reservoir. Furthermore, the separation of sites with higher MPs concentrations (Rabagão sites, which exhibited Good Ecological Potential) from sites with Moderate Ecological Potential (Aguieira sites) suggests that microplastic pollution may not always correlate directly with traditional water quality parameters, highlighting the need to include MPs in future monitoring programs.

## 4 Discussion

### 4.1 Ecological characteristics and human pressures on the reservoirs

The present study is the first to aim at characterizing and analysing MPs in Portuguese reservoirs, considering the characteristics and anthropogenic pressures in the hydrographic basin. In the Rabagão reservoir the human presence is mainly observed in areas dedicated to agriculture and pastures, predominantly traditional and family-based (POAAR, 2009; Cabecinha et al., 2009; Almeida et al., 2020). This low level of human interference is reflected in the analysis of the recorded pressures for this area, with the presence of a trout farm being the main pressure identified (Table 1). Overall, the reduced anthropogenic impact contributed to the classification of Good Ecological Potential recorded in this study (Table 2), consistent with the evaluations from the 3^rd^ planning cycles of the River Basin Management Plans (PGRH) under the WFD program (APA, 2023b).

Almeida et al. (2020) also assessed the water quality of the Rabagão reservoir and showed varying classifications for the physical and chemical parameters, ranging from Good or Superior to Moderate across the different seasons studied. In particular, the Moderate classification in summer contrasts with the Good classification obtained in the present study. Almeida et al. (2020) attributed this lower classification primarily to low dissolved O_2_ levels (mg/L and %) observed, similar to those recorded in the present study, where an O_2_ value was below the Excellent threshold, in Sum2 (Table 2). In the present water quality assessment of the Rabagão reservoir (Table 2), higher concentrations of NO_2_ and TSS had a more significant impact on the classification of Good Ecological Potential. Higher concentrations of nutrients in the water are often associated with agricultural and livestock practices (used as fertilizers), especially during rainy months due to soil leaching (Turner and Rabalais, 2003; Ramião et al., 2022). Therefore, despite the low agricultural pressure in Rabagão surrounding area (15.7%, Table 1), this phenomenon may explain some higher nutrient concentrations observed, even though they remain within the limits for Good Ecological Potential. Furthermore, the presence of a trout farm may be another factor influencing the nutrient and TSS load in the water body (Pulatsu et al., 2004). However, the Rabagão reservoir has been classified as oligotrophic or mesotrophic (Cabecinha et al., 2009; Almeida et al., 2020), with low to medium nutrient levels, resulting in moderate phytoplankton growth and moderate concentrations of chlorophyll *a*. The variation in this classification can be attributed to occasional human activities (e.g., effluent discharges, intensification of livestock, agricultural, or recreational activities) in addition to seasonality. The biological classification obtained in this study is consistent with previous information, as the chlorophyll *a* concentration in the summer samples consistently fell within the threshold for a “Good” classification, with only the concentration in the autumn samples exceeding this value. Indeed, although they do not pose a major risk for this reservoir, the reduction of microbiological and organic pollution, as well as precursors of eutrophication processes (e.g., high concentrations of nitrates, phosphorus, phosphorus compounds, chlorophyll *a*, and the occurrence of microalgae blooms) were identified as factors to be improved in the 2^nd^ cycle of the PGRH (APA, 2016b).

Aguieira reservoir presents a landscape predominantly composed of forests, mainly eucalyptus, acacias, and pines used for intensive forestry (POAA, 2005; Geraldes et al., 2016; Pinto et al., 2021a). Moreover, this reservoir is subject to high anthropogenic pressure, with a high number of WWTPs in the surrounding area (Table 1; Vasconcelos et al., 2011). Navigation support structures are also present, near the sampling sites, attracting large numbers of people, especially in summer, for recreational activities such as sailing, rowing, and using the area as river beaches (POAA, 2005). This high level of anthropogenic pressure is reflected in the Moderate classification obtained for most of the summer samples (Table 2), consistent with the last classification obtained in the 3^rd^ planning cycle of the PGRH under the WFD program (2022–2027; APA, 2023c).

Regarding the physical and chemical parameters, the classification was primarily influenced by high conductivity, temperature, and pH values, as well as high concentrations of PO_4_^3-^ and low transparency. These results are aligned with previous studies, particularly by Geraldes et al. (2016) with samples collected in the spring and summer of 2010 and 2011, and Pinto et al. (2021a) and Pinto et al. (2021b) with a study conducted in the spring and autumn of 2018–20. Beyond agricultural activities, which are identified in the 3^rd^ cycle of the PGRH (APA, 2023c) as one of the main sources of pollution to be controlled, forestry and WWTPs are also frequently associated with increased nutrient concentrations in aquatic ecosystems (Lee et al., 2019; Diogo et al., 2022). The 3^rd^ cycle of the PGRH (APA, 2023c) highlights nutrient pollution from wastewater in this reservoir and emphasizes the need for interventions in drainage and wastewater treatment systems to enhance the ecological quality of this aquatic ecosystem. Jarvie et al. (2006) found that punctual effluent discharges can have a greater impact on nutrient concentrations in water than agricultural activities, even in rural areas, based on data collected from 54 rivers in the United Kingdom. Excess of nutrients promotes microalgal proliferation, leading to increased chlorophyll *a* concentration and a decrease in dissolved oxygen and transparency, ultimately causing a loss of ecological quality (Carey and Migliaccio, 2009). Indeed, in the biological component analysis conducted in this study, high chlorophyll *a* concentrations were observed in samples from multiple sampling periods (Table 2). Furthermore, due to the influence of these varied pressures, the Aguieira reservoir has been classified as eutrophic, with high nutrient concentrations and frequent cyanobacterial blooms (Vasconcelos et al., 2011; Geraldes and Silva-Santos, 2013), which is consistent with the Moderate Ecological Potential classification obtained in this study.

### 4.2 Microplastic distribution and sources

The results obtained in this study regarding the evaluation of MPs in reservoirs confirm that heavily modified water bodies are indeed propitious environments for the accumulation of these emerging pollutants. In the two study reservoirs, the sampling site closest to the dam (Rb1 and Ag1) showed the highest concentration of MPs, in almost all sampling periods (Figure 2). Rb1 is also the site nearest to the aquaculture structure located in the Rabagão reservoir. Several studies have evidenced the link between aquaculture practices and plastic pollution in freshwater ecosystems, particularly due to the loss and disposal of materials such as fishing nets and buckets, as well as the construction of infrastructure and the transport and storage of aquatic species (FAO, 2017; Ding et al., 2021). In Ag1, in the Aguieira reservoir, several navigation support structures are present, where water sports are frequently practiced. These activities have also been widely documented as closely associated with the release of plastic particles into water bodies (e.g., Raposo et al., 2022). Prata et al. (2021), in a study evaluating the abundance of MPs across three zones with varying anthropogenic pressures in the Douro River (Portugal), observed a higher concentration of MPs near a dock and boat maintenance area compared to a rural zone and a wastewater effluent discharge area. Additionally, Ag1 is situated at the confluence of the three rivers feeding this reservoir, Mondego, Dão, and Criz. Haque et al. (2024) already observed higher concentrations of MPs in the confluence zone with the St. Lawrence River, in the assessment of the distribution of MPs in the Raquette River (United States of America). Similarly, Da Costa et al. (2023) reported higher concentrations of MPs in confluence areas of the Paraíba do Sul River basin (Brazil) compared to upstream locations, likely due to the role of tributaries in sediment and MPs transport from adjacent urban areas. Besides the factors mentioned, dams act as physical barriers to the flow of water and sediments, promoting the accumulation of MPs transported by rivers, as described for sediments and nutrients (Almeida et al., 2020; Pinto et al., 2021b). This factor can explain the higher concentration of MPs at sites closer to the dam (Rb1 and Ag1), consistent with findings from previous studies in reservoirs worldwide, both for surface water samples (Zhang et al., 2015; Min et al., 2023) and sediment samples (Queiroz et al., 2024; Haque et al., 2024).

Regarding the type classification, fibres, fragments, paints, and films were identified in both studied reservoirs. It is noteworthy that foam particles and pellets were not observed in the samples collected in this study. Rodrigues et al. (2018), in a study evaluating the presence and abundance of MPs in samples from the Antuã River (north-central Portugal), observed that foam particles and pellets were almost undetectable. Similarly, Rodrigues et al. (2020) did not detect these types of particles in water samples from the northwestern coastal zone of Portugal. Pellets, which are recognized as primary MPs are less frequent in aquatic ecosystems and their occurrence has been decreasing, partly due to legislation and regulatory measures aimed at reducing the use of single-use plastics (Nava et al., 2023; Union European, 2023). Conversely, particles such as fibres, fragments, paints, films, and foams are considered secondary MPs, resulting from the fragmentation and degradation of larger plastics (Golwala et al., 2021). These findings are consistent with the observation that secondary MPs are the most found in aquatic ecosystems (Nava et al., 2023), as also reported by Rodrigues et al. (2018), Rodrigues et al. (2019), and Rodrigues et al. (2020) in different Portuguese ecosystems, including the Antuã River, Douro estuary, and the northwestern coastal zone.

Fibres were the most frequently identified type of microplastic in both reservoirs (Figure 3A), aligning with the findings of Li et al. (2020), who reported that fibres are the most common microplastic type in freshwater ecosystems. Similarly, studies conducted in reservoirs worldwide under different anthropogenic pressures, such as Three Gorges (Di and Wang, 2018), Danjiangkou (Di et al., 2019; Lin et al., 2021), and Liujiaxia (Min et al., 2023) in China, Billings in Brazil (Queiroz et al., 2024), and Alqueva in Portugal (Raposo et al., 2022), consistently reported fibres as the predominant type of microplastic. Surface runoff and atmospheric deposition are examples of pathways through which plastic fibres from adjacent urban and industrial areas reach reservoirs (Min et al., 2023). Additionally, fishing and aquaculture materials represent direct sources of these pollutants in aquatic environments (Di et al., 2019). However, plastic fibres have also been detected in lakes and reservoirs located in remote areas with minimal human activity (Nava et al., 2023). Textiles are recognized as one of the main contributors to the presence of this type of microplastic in aquatic ecosystems, as fibres are released during clothing washes and cannot be totally retained by WWTPs (Li et al., 2020; Min et al., 2023). Furthermore, recreational activities during the bathing season may also contribute to increased fibre concentrations in reservoirs (Nava et al., 2023), associated with clothing (e.g., swimwear) and equipment like surfboards, as demonstrated by Gao et al. (2021) in their study on the seasonal distribution and characterization of MPs on Qingdao beaches (China). All potential fibres sources identified in previous studies were also observed in this study (Table 1), with aquaculture practices being a prominent source in the Rabagão reservoir. In contrast, the Aguieira reservoir exhibited a greater diversity and number of pressures, particularly due to the high concentration of nearby WWTPs.

Paint particles were the second most frequently identified type of microplastic in both reservoirs (Figure 3A). Although less studied, this type of microplastic has gained increasing attention in recent years, largely due to its association with the abrasion of boat and ship surfaces during use and maintenance (Turner et al., 2022). Prata et al. (2021) observed high concentrations of paint particles near a marina on the Douro River and emphasized the value of chemical characterization in identifying these particles more accurately. In both reservoirs, the frequent use of boats and other aquatic infrastructure, especially during the bathing season, may have contributed to these results. Additionally, paint particles resulting from the degradation of structures in the surrounding terrestrial area can be transported to aquatic ecosystems through precipitation or wind (Gaylarde et al., 2021).

Contrary to the findings of this study, Nava et al. (2023) identified fragments, rather than paint particles, as the most frequently observed MPs after fibres, in a study of 38 lentic ecosystems worldwide. Scherer et al. (2020), in their evaluation of MPs in the Elbe River (Germany), found that fragments were the predominant typology in sediment samples, while fibres were more abundant in water column. Fragments have a lower surface-to-volume ratio and tend to be denser, leading to faster sinking (Scherer et al., 2020). Since this study only analysed subsurface water samples, this could explain the lower abundance of fragments observed. Additionally, the lower frequency of smaller MPs observed in this study may be explained by their tendency to associate with biofilms, which increases their density and causes them to sink. Biofilm formation on particles is particularly prevalent in reservoirs due to the longer water residence time (Liu S. et al., 2022). Furthermore, biofilm-covered particles are more likely to be ingested by organisms, as biofilms enhance their palatability (Sucharitakul et al., 2021), which may further reduce their detectability in the water column.

Concerning colour, blue and black are the most identified colours of MPs in reservoirs worldwide (Di et al., 2019; Min et al., 2023), as well as in other freshwater ecosystems in Portugal (Rodrigues et al., 2018). A lower percentage of transparent MPs recorded (maximum of 28% in Ag3 in Aut), compared to other studies (Di et al., 2019; Li et al., 2021), may be attributed to the relatively lower intensity of fishing activities in both study areas. Several studies have identified fishing activities as one of the primary sources of transparent MPs, alongside plastic bags and food packaging (e.g., Di et al., 2019). Consistent with Haque et al. (2024), some MPs exhibited faded colours (partially transparent and partially coloured), indicating their prolonged presence in the aquatic ecosystem and exposure to degradation processes. Other MP colours are associated with diverse sources, including the breakdown of coloured plastics used in everyday life, textile fibres released through WWTPs effluents, coloured paints from boats and nearby structures, black agricultural films, tire wear, among others (Di et al., 2019; Li et al., 2021).

Regarding the chemical analysis of MPs, the ATR-FTIR technique proved to be a fast, accurate, relatively sensitive method that does not require complex sample preparation, making it a valuable tool for identifying the potential MPs sources. The results showed the predominance of PE particles in both reservoirs (Figure 4), trend observed in various regions around the world (e.g., Rodrigues et al., 2018; Scherer et al., 2020; Li et al., 2021). Similarly, in the study conducted by Rodrigues et al. (2018) in the Antuã River, Portugal, PE and PP were found to be the most prevalent polymers, both in water and sediment samples. Scherer et al. (2020) identified PE as the most common polymer in the Elbe River, Germany, while Li et al. (2021), in a review of several aquatic ecosystems in China, also found PE to be the predominant polymer. These results suggest a strong correlation between the extensive use of polymers like PE and their high presence in aquatic systems, confirming the persistent and widespread nature of this polymer across regions with diverse geographic characteristics and levels of anthropogenic activity. PE is one of the most widely produced polymers, found in everyday items such as ropes, clothing, toys, and disposable packaging (Min et al., 2023; Nava et al., 2023). Due to its short lifecycle and improper disposal, PE is a major contributor to pollution in aquatic ecosystems (Nava et al., 2023). Its versatile usage helps explain the significant presence of these MPs in the studied reservoirs, which are in areas with varying degrees of anthropogenic pressures (Figure 1). Moreover, Di and Wang (2018) observed the prevalence of lower-density polymers (e.g., low-density PE) in water samples from the largest reservoir in China, compared to sediment samples. This occurs because lower-density MPs tend to float, while higher-density MPs (e.g., PVC, PET) tend to sink and deposit in sediments. PVC and PET are also among the most produced non-fibrous plastics globally (Geyer et al., 2017). Like PE, PET is widely used in disposable packaging, usually in its amorphous form. Both PET and PE are plastics with short lifecycles, often discarded improperly, which explains their abundance in water bodies. As for PVC, although it is also highly produced, it is less frequently identified in studies since it is primarily used in construction materials for what its fragmentation and release into the aquatic environment occur more sporadically and slowly (Geyer et al., 2017; Bordós et al., 2019; Rani-Borges et al., 2022). On the other hand, PES is the most used polymer in the form of fibres, primarily linked to the textile industry. The washing of clothing releases large quantities of PES fibres, and effluent discharges accumulating these MPs are the main entry route into water bodies (Bordós et al., 2019; Min et al., 2023). Additionally, NY, a generic term for polymers made of polyamides, is widely used in clothing, as well as in non-fibrous forms for disposable packaging, rubber, automotive parts, and electrical equipment (Fan et al., 2021). Finally, PAV is a polymer used in adhesives, coatings, and paints (Florido and Lordsleem Jr, 2023). SAN, PS, NBR, PSU, SBR, and PP were the least abundant polymers and were observed for only one of the reservoirs.

It is important to note that, although less common than methods such as manta nets or pumping systems, the use of 1 L glass bottles for sub-surface water sampling has proven to be effective for MPs assessment based on the aims of this study. Considering that reservoirs are lentic environments and the study focused exclusively on sub-surface water, this point-sampling approach has shown to be suitable for such conditions. Moreover, while manta nets are limited by mesh size—typically excluding particles smaller than 300 µm—the grab method allows for the collection of a wider range of particle sizes, with the only limitation being the pore size of the filters used during laboratory filtration (Green et al., 2018; Prata et al., 2020). This is ecologically relevant, especially considering that smaller particles tend to pose greater risks to organisms (Prata et al., 2024). Additionally, the sampled volume is more precise, reducing potential errors (Green et al., 2018). The grab method is also rapid, easy to apply in the field, and does not require any energy source or expensive equipment, enabling a higher number of replicates and thus enhancing statistical robustness and representativity (Barrows et al., 2017). These characteristics are particularly relevant, as this study aimed to emphasize the importance of incorporating MPs analysis into continuous monitoring programs under directives such as the WFD. Furthermore, since results are expressed in MPs/L, they are comparable to those obtained using other sampling methods when properly normalized, especially given that the post-sampling analytical procedures used here are consistent with those most reported.

The characterization and distribution of MPs observed in this study followed a pattern consistent with findings from other studies conducted in aquatic ecosystems worldwide (e.g., Di and Wang, 2018; Rodrigues et al., 2018; Haque et al., 2024; Queiroz et al., 2024). The main difference observed was the variation in MP concentrations between the two reservoirs, with less MPs identified in the Aguieira reservoir compared to the Rabagão reservoir. The Rabagão reservoir features a more natural landscape, with less human activity and fewer man-made pressures. This is reflected in its higher ecological classification compared to the Aguieira reservoir at all sampling sites during the summer. On the other hand, the Aguieira reservoir has more human influence, including artificialized land use and a greater variety of anthropogenic pressures, resulting in lower ecological scores according to the WFD. However, despite its better ecological classification, the Rabagão reservoir showed higher concentrations of MPs. Among the pressures identified in this study area, aquaculture appears to be the most significant contributor to microplastic pollution, with agricultural practices and recreational activities also playing a role. Nevertheless, these pressures alone do not seem sufficient to explain the higher concentrations of MPs compared to the Aguieira reservoir, which experiences higher anthropogenic pressures. This indicates that the transport, accumulation, and retention of MPs in freshwater systems are influenced by complex factors beyond just human activity. For instance, although we do not have access to residence times of the water in this reservoir for the studied period, Rabagão reservoir has a history of longer residence times than the Aguieira reservoir, which may influence the amount of MPs accumulated in this reservoir. Cabecinha et al. (2009) reported mean residence times between 1996 and 2004 of 594.12 days for Rabagão and 50.59 days for Aguieira. Moreover, Rabagão reservoir receives water from other upstream reservoirs, which may themselves contribute with additional sources of pollution from their surrounding environments. Additionally, several parameters were determined according to WFD, since various studies have demonstrated interactions between them and MPs. Variations in pH, temperature, and conductivity can affect MPs’ surface properties, aggregation, and buoyancy, influencing their mobility and vertical distribution (Buwono et al., 2021). Dissolved oxygen, water transparency, nutrient concentrations, and chlorophyll *a* reflect biological productivity and suspended particulate matter, which can promote MPs’ attachment or biofilm formation, thereby affecting their transport, buoyancy, and residence time (Chen et al., 2020; Zhao et al., 2024). High BOD_5_ and TSS indicate elevated organic and particulate loads that may act as carriers or sinks for MPs. Conversely, MPs themselves can reduce water transparency (Buwono et al., 2021). However, these interactions may vary depending on the ecosystem under study, and, given the results obtained, highlighted in Figure 6, these conventional water quality indicators may not adequately capture the impact of emerging pollutants like MPs. The analysis of other environmental factors, such as hydrodynamics, sedimentation processes, biological interactions, and the presence of specific aquatic structures, which can influence the fate and distribution of MPs in reservoirs (Shen et al., 2023; Guo et al., 2024), would lead to a better understanding of the results obtained. Besides that, this study was limited to surface water samples, which provide valuable information on current pollution sources and ongoing inputs, and are particularly relevant for trophic transfer and potential implications for public health. Nevertheless, this limitation may affect the representativeness of the results due to the potential vertical stratification of MPs in the water column and sedimentation processes. Future research should consider sampling at multiple depths to improve the robustness and comprehensiveness of the findings. Overall, this apparent discrepancy between the water quality of the studied reservoirs and the observed concentration of MPs emphasizes the importance of implementing monitoring and evaluating these pollutants in freshwater bodies, particularly in reservoirs, to better understand the scope of their impact on aquatic ecosystems.

## 5 Conclusion

Our results revealed that MPs were identified in all sampling sites and periods analysed, demonstrating that even in ecosystems with better ecological water quality, such as Rabagão, which appear less impacted, these pollutants are already present. A total of 1,658 MPs were identified in the Aguieira reservoir and 5,862 in Rabagão, with fibres between 0.1 and 0.5 mm being the predominant form, mainly in blue, black, and grey. MPs concentration was higher in the sampling site closest to the dam in both study areas. The characterization of the study areas regarding land use and land cover, and anthropogenic pressures highlighted a less natural landscape and a higher number of pressures in the area surrounding the Aguieira reservoir, correlating with its classification of Moderate Ecological Potential, primarily attributed to high concentrations of nutrients. In contrast, the Rabagão reservoir, which showed a higher concentration of MPs, was characterized by a more natural landscape and lower anthropogenic pressure. Potential sources of MPs include aquaculture, WWTPs discharges, water sports, and recreational activities, especially during summer. ATR-FTIR analysis identified PE as the most common polymer, along with other detected polymers such as PET, PES, NY, PVC, and PVA, providing insights into the potential sources of these particles in the studied reservoirs.

The presence of MPs in high concentrations in water bodies of extreme social importance, such as reservoirs, poses a risk to both human health and the quality of these ecosystems. Assessing a larger number of reservoirs, with varying pressures and ecological quality classifications, would provide a deeper understanding and emphasize the importance of incorporating MPs monitoring into ecological quality assessments as a complement to the WFD. Additionally, to better understand the results obtained, it would be important to analyse the concentration of MPs in the sediments of these reservoirs, as it may differ from the water column. MPs sinking can be influenced by factors such as biofilm formation and hydrodynamics, which could lead to different results among the reservoirs. Furthermore, it is crucial to standardize the methods used for MPs research to enable more accurate comparisons between studies and facilitate data sharing, which will support the implementation of more effective measures to address MPs pollution.

## Data Availability

The raw data supporting the conclusions of this article will be made available by the authors, without undue reservation.
